# Biodegradation and detoxification of textile azo dyes by bacterial consortium under sequential microaerophilic/aerobic processes

**DOI:** 10.17179/excli2014-642

**Published:** 2015-01-29

**Authors:** Harshad Lade, Avinash Kadam, Diby Paul, Sanjay Govindwar

**Affiliations:** 1 Department of Environmental Engineering, Konkuk University, Seoul-143-701, Korea; 2 Department of Environmental Engineering, Kyungpook National University, Daegu-702-701, Korea; 3 Department of Biochemistry, Shivaji University, Kolhapur-416004, India

**Keywords:** Azo dyes, P. rettgeri strain HSL1, Pseudomonas sp. SUK1, bacterial consortium, decolorization, biodegradation, sequential microaerophilic/aerobic process, detoxification

## Abstract

Release of textile azo dyes to the environment is an issue of health concern while the use of microorganisms has proved to be the best option for remediation. Thus, in the present study, a bacterial consortium consisting of *Providencia rettgeri* strain HSL1 and *Pseudomonas sp*. SUK1 has been investigated for degradation and detoxification of structurally different azo dyes. The consortium showed 98-99 % decolorization of all the selected azo dyes viz. Reactive Black 5 (RB 5), Reactive Orange 16 (RO 16), Disperse Red 78 (DR 78) and Direct Red 81 (DR 81) within 12 to 30 h at 100 mg L^-1^ concentration at 30 ± 0.2 °C under microaerophilic, sequential aerobic/microaerophilic and microaerophilic/aerobic processes. However, decolorization under microaerophilic conditions viz. RB 5 (0.26 mM), RO 16 (0.18 mM), DR 78 (0.20 mM) and DR 81 (0.23 mM) and sequential aerobic/microaerophilic processes viz. RB 5 (0.08 mM), RO 16 (0.06 mM), DR 78 (0.07 mM) and DR 81 (0.09 mM) resulted into the formation of aromatic amines. In distinction, sequential microaerophilic/ aerobic process doesn’t show the formation of amines. Additionally, 62-72 % reduction in total organic carbon content was observed in all the dyes decolorized broths under sequential microaerophilic/aerobic processes suggesting the efficacy of method in mineralization of dyes. Notable induction within the levels of azoreductase and NADH-DCIP reductase (97 and 229 % for RB 5, 55 and 160 % for RO 16, 63 and 196 % for DR 78, 108 and 258 % for DR 81) observed under sequential microaerophilic/aerobic processes suggested their critical involvements in the initial breakdown of azo bonds, whereas, a slight increase in the levels of laccase and veratryl alcohol oxidase confirmed subsequent oxidation of formed amines. Also, the acute toxicity assay with *Daphnia magna* revealed the nontoxic nature of the dye-degraded metabolites under sequential microaerophilic/aerobic processes. As biodegradation under sequential microaerophilic/aerobic process completely detoxified all the selected textile azo dyes, further efforts should be made to implement such methods for large scale dye wastewater treatment technologies.

## Introduction

Textile dyes are chemicals of complex aromatic structures designed to resist the impact of detergents, sunshine and temperatures (Nigam et al., 1996[37]). They are chemically and photochemically stable and are extremely persistent in natural atmospheres. The world-wide annual production of synthetic textile dyestuff has been estimated to be over 1 x 10^6^ ton (Pandey et al., 2007[39]). More than 10,000 different commercially available dyes are used in textile industry for dyeing and printing purposes (Meyer, 1981[34]; Kadam et al., 2011[23]). The fixation rates of several textile dyes are not 100 % and around 30-70 % of the amount of dyestuff used to get eliminated into effluent during the wet processing operations (Khaled et al., 2009[23]; Bumpus, 1995[4]). The estimated dyes concentration in the textile effluent has been reported to be in the range of 10-200 mg L^-1^ (Kadam et al., 2011[23]). 

Textile dyes are chemically diverse in nature and are broadly divided into azo, reactive, triphenylmethane, heterocyclic, polymeric structures, etc (Cheunbarn et al., 2008[9]). Among these various types, the azo dyes constitute about 70 % and are used widely for dyeing purposes. This makes them the largest and most important group of synthetic colorants released into the environment (Ambrósio and Campos-Takaki, 2004[1]). In order to satisfy the standards necessary for industrial applications like stability and long lasting of colorants, the azo dyes are manufactured with various colors, molecular structures and resistance to attenuation upon exposure to sunlight, water and several chemicals (Correia et al., 1994[12]). Azo dyes are of concern in textile wastewater treatment because its very small amount in water (10-50 mg L^-1^) is highly visible and aromatic amines that occurred from azo reduction are suspected to be carcinogenic and have toxic effect on organisms (Wong and Yu, 1999[60]). The structures of azo dyes consist of coupling of diazotized amine with either associated amine or a phenol and additionally possess an azo bond (N = N). The discharge of such dyes in natural ecosystem results into conversion of azo group to aromatic amines and further bioaccumulation of such products could result in toxic impact on aquatic life and even carcinogenic and mutagenic effect on humans (Banat et al., 1996[3]; Pinheiro et al., 2004[43]). Azo dyes are known to inhibit the tyrosinase enzyme which may leads to inhibition of melanin synthesis and results in hypopigmentation (Dubey et al., 2007[13]). The color of dyestuff in effluent interrupts the aquatic environment by reducing light penetration, gas solubility and interference of phytoplankton’s photosynthesis (Sharma, 2009[49]). In addition, the discharge of untreated effluent into usable water resources like rivers and lakes alters pH scale and will increase BOD, COD and TOC values (Lade et al., 2012[27]). 

The environmental legislation about the appearance of color in discharged effluent has forced the industries to treat dyestuff containing effluent themselves (Robinson et al., 2001[44]). Additionally, limited supply and increasing cost of water for industrial sector has made the treatment and reuse of dyeing effluent mandatory to avoid environmental pollution and reduce production cost. A variety of typical physico-chemical strategies such as coagulation, flocculation, activated carbon adsorption and reverse osmosis technique are used for the removal of color from textile effluent. However, excessive use of chemicals in such conventional treatments produces large amount of sludge and does not remove all dyes, thus preventing recycling of treated effluent. In addition, excess use of chemicals requires high cost and results into secondary pollution (Jadhav et al., 2007[21]). These methods can only transfer the dyestuff from one phase to another leaving the problem essentially unsolved. Alternatively, the genetic diversity and metabolic versatility of microorganisms makes them a biological option of treatment to remediate the pollution caused by dyes. Thus, the utilization of microorganisms for degradation and detoxification of dyes changing into an important technique because it is cost effective, eco-friendly and produces less amount of sludge (Banat et al., 1996[3]; Robinson et al., 2001[44]).

Microorganisms able to degrade azo dyes under microaerophilic conditions are known as azo linkages are easily reduced in such conditions (Tan et al., 1999[54]). Few conventional aerobic textile wastewater treatments are reported as not able to degrade azo dyes (Shaul et al., 1991[50]). Based on the available literature, it can be concluded that the microbial decolorization of azo dyes is more effective in sequential microaerophilic/aerobic and aerobic/microaerophilic processes (Franciscon et al., 2009[15]; Waghmode et al., 2011[57], 2012[58]). As in many cases the degradation metabolites produced in single microaerophilic condition are aromatic amines, which are toxic or even more toxic than parent azo dyes, thus require subsequent aerobic stage for their degradation (Hu, 2001[19]). It has been demonstrated that reductive enzymes break down azo bond of azo dyes under microaerophilic conditions while oxidative enzyme works better at aerobic conditions which allows its possible decolorization and further detoxification of formed amines (Zimmerman et al., 1982[61]; Duran and Esposito, 2000[14]). Therefore sequential microaerophilic/aerobic processes of azo dyes degradation become a promising approach for detoxification of textile effluent. 

In this context, the present study reports the decolorization of four structurally different textile azo dyes by a bacterial consortium consisting of* P. rettgeri* strain HSL1 and *Pseudomonas sp*. SUK1 under microaerophilic, aerobic, sequential aerobic/microaerophilic and microaerophilic/aerobic processes. The optimization of physico-chemical parameters for enhanced decolorization of selected azo dyes was carried out by one parameter at a time approach. The formation of aromatic amines was analyzed spectrophotometrically, whereas the reduction in total organic carbon was estimated using the TOC analyzer under various incubation processes. Activities of dye degrading reductive and oxidative enzymes were determined by standard assays. The environmental impact of azo dyes and its degraded metabolites was assessed by monitoring the acute toxicity to *Daphnia magna*.

## Material and Methods

### Textile dyestuff and chemicals

The textile azo dyes viz. C.I. Reactive black 5 (RB 5), C.I. Reactive orange 16 (RO 16), C.I. Disperse red 78 (DR 78) and C.I. Direct red 81 (DR 81) were kindly provided by Mahesh Textile Processors, Ichalkaranji, India. The structural information of dyes is shown in Table 1(Tab. 1). Veratryl alcohol, 2,2’-Azino-bis (3-ethylbenzothiazoline-6-sulfonic acid) (ABTS), methyl red, nicotinamide adenine dinucleotide (NADH), dichlorophenol indophenols (DCIP) and dehydrated microbiological medium nutrient broth (NB) were obtained from HiMedia Laboratories Pvt. Ltd., Mumbai, India. Chloranil, Dimethylformamide and Aniline-2-sulfonic were purchased from Sigma-Aldrich (St. Louis, MO, USA). 

### Bacterial strains and culture conditions

*P. rettgeri* strain HSL1 (Genebank accession no. JX853768.1) isolated from textile effluent contaminated soil was selected based on its capacity to degrade textile azo dye. Other bacterium* Pseudomonas sp*. SUK1 (Genebank accession no. EF541140) previously isolated from textile dye contaminated site and known for textile effluent decolorization was used to develop consortium (Kalyani et al., 2008[24], 2009[25]). The stock cultures of both the bacterial strains were maintained at 4 ± 0.2 °C on nutrient medium slants containing 1 % textile effluent to retain their dye decolorizing abilities.

### Pre-enrichment conditions

Both the bacterial cultures *P. rettgeri* strain HSL1 and *Pseudomonas sp*. SUK1 were individually grown in 250 ml Erlenmeyer flask containing 100 ml of nutrient broth having composition g L^-1^ of sodium chloride 5.0, beef extract 1.5, yeast extract 4.0 and peptic digest of animal tissue 5.0. The initial pH of the medium was adjusted to 7.0 with 0.1 M HCL and/or 0.1 M NaOH, autoclaved at 121 °C for 20 min, and separately inoculated with 100 µl of 24 h old active cultures of bacterial strains. The enrichment was carried out for 24 h at 30 ± 0.2 °C under microaerophilic conditions and then used as inoculum for further degradation studies.

### Optimization of decolorization conditions

Decolorization experiments were conducted in 250 ml Erlenmeyer flask containing 100 ml of individual pre-enriched *P. rettgeri* strain HSL1, *Pseudomonas sp*. SUK1 and equal amounts of both the cultures (50 ml of each) as bacterial consortium. The decolorization efficiency of individual cultures and bacterial consortium was investigated with 100 mg L^-1^ dyes concentration, initial broth pH of 7.0 (adjusted after pre-enrichment) and 30 ± 0.2 °C of incubation temperature under microaerophilic conditions. The process parameters optimization for all the azo dyes decolorization by bacterial consortium were performed in microaerophilic conditions by one parameter at a time approach viz. initial pH of pre-enriched broth (3 to 12), incubation temperature (20, 30, 37, 40, 50 ± 0.2 °C) and initial dye concentration (50 to 250 mg L^-1^).

### Decolorization at various processes

For the sequential aerobic/micro-aerophilic decolorization study, the azo dyes (100 mg L^-1^) containing bacterial consortium broths were incubated at aerobic stage for the first half time as required in complete decolorization under microaerophilic condition and then transferred to microaerophilic stage for subsequent decolorization for the next remaining time. Here, the aerobic process is defined as the incubation on orbital shaker at 120 rpm. The same procedure was followed for decolorization under sequential microaerophilic/aerobic processes. Influence of single and sequential incubation stages on decolorization performance and formation of aromatic amines by bacterial consortium were investigated at optimized conditions (Waghmode et al., 2011[57]; Franciscon et al., 2009[15]). 

At defined time of intervals, the aliquots of culture supernatant (3 ml) were withdrawn and suspended particles removed by adding equal volume of methanol followed by centrifugation at 7500 x g for 15 min at 4 ± 0.2 °C. The resulted supernatant was assayed spectrophotometrically by measuring the decrease in absorbance at respective dyes λmax (UV-vis spectrophotometer, Hitahi U-2800; Hitachi, Tokyo, Japan). The control flasks as without dye or bacterial cultures were also tested under the same conditions. If otherwise mentioned, all decolorization experiments were performed in triplicate and decolorization activity was expressed in terms of percent decolorization using the formula: 


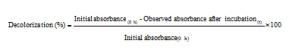


### Determination of aromatic amines 

The aromatic amines formed in decolorized broth were determined spectrophotometrically as per the method of Marik et al. (2003[33]). Briefly, the broth samples were taken after decolorization under microaerophilic, aerobic, sequential aerobic/microaerophilic and microaerophilic/aerobic processes, frozen and freeze-dried in Upright Freeze Dryer Model: FDU5003/8603 (Operon Co. Ltd, Korea). The freeze-dried samples (5 mg) were then dissolved in 5 ml of a 0.4 % chloranil solution in dimethylformaminde (DMF) and heated to 100 ± 0.2 °C for 5 min. The absorbance was measured spectrophotometrically at 560 nm. A calibration curve of aniline-2-sulfonic acid as a model amine product of azo dyes reduction was prepared and the concentration of sample amines was calculated in mM. The pre-enriched cultures in NB without dyes were used as control. 

### Total organic carbon measurement

The presence of organic carbon in the culture broth containing dyes was measured by estimating the total organic carbon (TOC) under different incubation conditions using the TOC analyzer (Hach DR 2700 spectrophotometer, Hach Co., USA) (Lade et al., 2012[27]). The decolorized broth cultures were centrifuged at 7500 x g for 15 min and filtered through 0.45 µm pore size filter to remove bacterial cell debris. The removal ratio of TOC was calculated as follows:


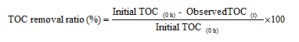


where TOC (0 h) and TOC (t) are the initial TOC value at (0 h) and the TOC value after particular reaction time (t), respectively.

### Enzyme activities

Extraction of enzymes after decolorization of dyes by bacterial consortium under sequential microaerophilic/aerobic conditions was carried out as per the procedure described earlier (Phugare et al., 2011[42]). Briefly, the bacterial cells were harvested by centrifugation at 7500 x g for 15 min in cold condition (4 ± 0.2 °C) and considered as test sample for determination of extracellular enzyme activities. The resulted cell pellets were suspended in 50 mM potassium phosphate buffer (pH 7.4), homogenized and sonicated by giving 7 strokes of 30 s each for 2 min interval based on 50 amplitude output at 4 ± 0.2 °C (Sonics-vibracell ultrasonic processor). These sonicated cells were further centrifuged at 7500 x g for 15 min at 4 ± 0.2 °C and supernatant was used as the source of intracellular enzymes. Similar protocol was followed to quantity the enzyme activities of control broth. The developed consortium without adding dyes was considered as control. 

Activities of both oxidative as well as reductive enzymes such as laccase, veratryl alcohol oxidase, azoreductase and NADH-DCIP reductase were assayed using UV-vis spectrophotometer (Hitachi U-2800, Japan). Laccase activity was determined by measuring the oxidation of 2’-Azino-bis-(3-ethylbenzothiazoline-6-sulfonic acid) at 420 nm (_ξ420_nm= 36000 (M cm)^-1^) (Wolfenden and Willson, 1982[59]). Veratryl alcohol oxidase activity was determined by monitoring the formation of veratraldehyde at 310 nm (_ξ310_nm= 9300 (M cm)^-1^ (Jadhav et al., 2009[22]). Azoreductase assay was performed in a reaction mixture of 2.0 ml containing the 4.45 µM of Methyl red and 0.2 ml of enzyme solution in 50 mM potassium phosphate buffer (pH 7.5). The reaction was started by adding 100 µM of NADH and decrease in color absorbance due to the reductive cleavage of methyl red was monitored at 430 nm (_ξ430_nm= 23360 (M cm)^-1^) (Chen et al., 2005[7]). The NADH–DCIP reductase activity was determined in a reaction mixture of 5.0 ml containing 25 µM DCIP and 0.2 ml of enzyme solution in 50 mM potassium phosphate buffer (pH 7.5). From this, 2.0 ml reaction mixture was assayed by adding 250 µM NADH and reduction in DCIP was measured at 590 nm (19 mM^-1^ cm^-1^) (Salokhe and Govindwar, 1999[45]). All experiments were run in triplicate at 30 ± 0.2 °C, average rates were calculated and one unit of enzyme activity was defined as a change in absorbance unit min^-1^ mg of protein^-1^. The protein content was determined by using the method of Lowry et al. (1951[30]) with bovine serum albumin as standard.

### Acute toxicity studies

The toxicity of azo dyes and its degraded metabolites were determined by acute toxicity test with *Daphnia magna* as described previously (Franciscon et al., 2009[15],[16]). Briefly, the dyes decolorized broth after treatment with bacterial consortium were centrifuged at 7500 x g for 20 min in cold condition, supernatant was collected and sterilized by passing through 0.45 µm pore size filter. The resulted filtrate was then used to perform sensitivity tests using 6 to 24 h old neonates. Each dye filtrate was then diluted to 25, 50, 75 and 100 % in an Erlenmeyer flask and 5 organisms were added. The tests were carried out at 20 ± 0.2 °C for 48 h in the absence of light and number of immobile organisms was counted after exposing to light for 20 seconds. Tests were carried out in triplicate for each concentration and control in distilled water. 

### Statistical analysis

Statistical analysis was carried out using the software SPSS 17.0 (SPSS, Chicago, IL, USA). The significance of variance was analyzed by one-way ANOVA with Tukey-Kramer multiple comparison test. 

## Results and Discussion

### Decolorization of textile azo dyes

**Figure 1:**
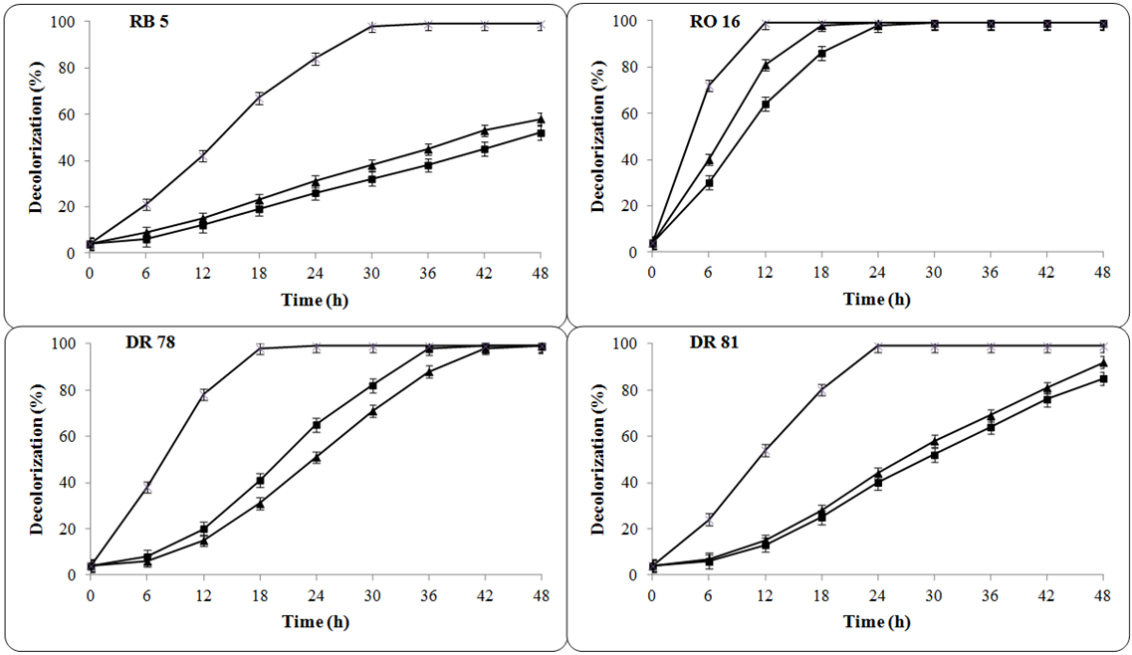
Decolorization of four azo dyes (100 mg L^-1^) by individual P. rettgeri strain HSL1 (-■-), *Pseudomonas sp.* SUK1 (-▲-) and its consortium (-x-) under microaerophilic conditions. The percent decolorization was measured at respective dyes λmax after different time of intervals at 30 ± 0.2 °C incubation temperature. Data points indicate the mean of three independent replicates, standard error of mean is indicated by error bars.

The chemical structural differences in textile dyes due to the substitution of various functional groups on aromatic base greatly influence their decolorization rates (Chivukula and Renganathan, 1995[10]; Pasti-Grigsby et al., 1992[40]). Thus, the bacterial consortium consisting of previously isolated dyes degrading bacteria *P. rettgeri* strain HSL1 and *Pseudomonas sp. *SUK1 has been developed and investigated for decolorization of structurally different azo dyes under microaerophilic and aerobic conditions. The results of the decolorization study indicate that both the individual cultures and its consortium were failed to completely decolorize selected dyes (100 mg L^-1^) under aerobic conditions and only 12-22 % performance was achieved within 48 h. On the other hand, individual cultures *P. rettgeri* strain HSL1 and *Pseudomonas sp*. SUK1 were able to completely decolorize two azo dyes viz. RO 16 (24 and 18 h) and DR 78 (36 and 42 h) and partially decolorize another dyes viz. RB 5 (52 and 58 %) and DR 81 (85 and 92 %) in microaerophilic conditions within 48 h respectively (Figure 1(Fig. 1)). 

All the dyes showed different extent of decolorization by both the cultures as the variation in chemical structural characteristic significantly affects the biotransformation process (Senan and Abraham, 2004[48]; Moosvi et al., 2005[35]). Decolorization of RO 16 and DR 78 appears in less time by both the cultures as compared to incomplete removal of RB 5 and DR 81 even at the end of 48 h of incubation period suggesting the inability of individual cultures to decolorize two dyes. However, bacterial consortium showed complete decolorization of all the four azo dyes viz. RB 5 in 30 h, RO16 in 12 h, DR 78 in 18 h and DR 81 in 24 h at same incubation conditions. The rate of decolorization by bacterial consortium was very high from the very beginning of the experiment and it has completely decolorized all the selected azo dyes. These results suggest that, the average decolorization rate of the bacterial consortium was significantly higher than that observed for individual bacterial cultures. In consistent with these findings, the higher decolorization rate of reactive azo dye Green HE4BD by developed microbial consortium GR of *P. vulgaris* and *M. glutamicus* cultures was reported when compared with its constituent pure strains (Saratale et al., 2010[46]). In addition, a fungal-bacterial consortium-AP consisting of *A. ochraceus* NCIM 1146 fungi and *Pseudomonas sp*. SUK1 bacteria and a bacterial consortium SDS consisting of *Providencia sp.* SDS and *Ps. aeuroginosa* strain BCH were reported for enhanced decolorization of azo dyes Rubine GFL and Red HE3B than its individual cultures (Lade et al., 2012[27]; Phugare et al., 2011[41]). The enhanced decolorization performance by bacterial consortium might be due to the synergistic reactions of individual strains in the consortium (Chen and Chang, 2007[6]). It is known that, in microbial consortium the individual strains may attack the dye molecule at various positions or may utilize the degradation metabolites generated by co-existing strains for further degradation which results in enhanced decolorization of dyes (Moosvi et al., 2007[36]; Asgher et al., 2007[2]).

### Optimization of decolorization conditions

**Figure 2:**
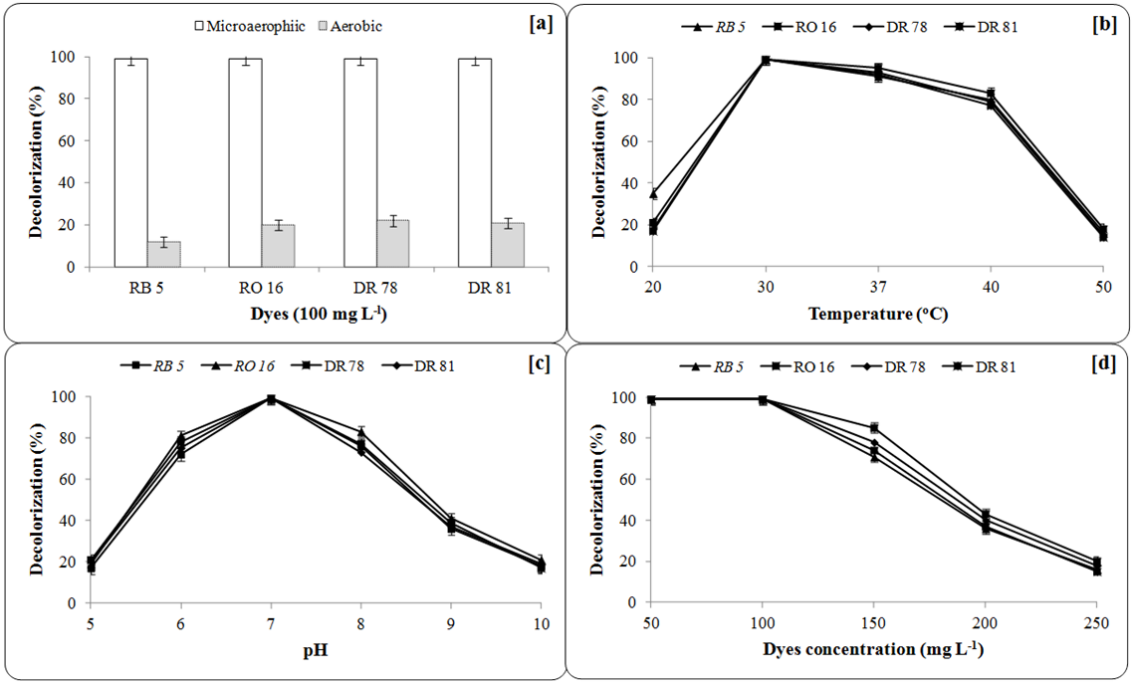
Effect of [a] Microaerophilic and shaking incubation, [b] incubation temperature, [c] initial broth pH and [d] dyes concentration on decolorization of azo dyes by bacterial consortium. Decolorization was measured after 30 h for RB5, 12 h for RO 16, 18 h for DR 78 and 24 h for DR 81. Data points indicate the mean of three independent replicates, standard error of mean is indicated by error bars.

Decolorization performance of bacteria has been known to be greatly influenced by various environmental conditions. For the enhancement of decolorization rate and to design an affordable treatment technology for textile effluent containing structurally different azo dyes, the optimization of decolorization conditions has been carried out. The complete and enhanced decolorization of all azo dyes (100 mg L^-1^) was observed within 12-30 h by bacterial consortium under microaerophilic conditions while only 12 % RB 5, 20 % RO 16, 22 % DR 78, and 21 % DR 81 dye removal performance was achieved under shaking conditions within the same time (Figure 2a(Fig. 2)). Hence, the microaerophilic conditions were adopted to optimize pH, temperature and dye concentration for enhanced degradation studies.

The result of the azo dyes decolorization by bacterial consortium at 100 mg L^-1^ concentration suggested that, 30 ± 0.2 °C was the best temperature for enhanced decolorization of all dyes in microaerophilic conditions (Figure 2b(Fig. 2)). Further increase or decrease in the temperature resulted in reduction of decolorization performance. Moosvi et al. (2007[35]) reported that a bacterial consortium JW-2 consisted of *Paenibacillus polymyxa*, *Micrococcus luteus *and *Micrococcus sp. *exhibited good decolorization ability of Reactive Violet 5R in the temperature range from 25 to 37 °C. The pH study showed that, bacterial consortium was able to decolorize all dyes at broad range of initial broth pH, however the optimum pH was found to be 7.0 for pre-enriched broth. Decrease in decolorization performance was observed at lower pH (3-6) and higher pH (8-12) (Figure 2c(Fig. 2)). Phugare et al. (2011[41]) found that pH 7.0 was optimum for enhanced decolorization of azo dye Red HE3B by a bacterial consortium consisting of *Providencia sp.* SDS and *Ps. aeuroginosa* strain BCH. The pH is directly associated with the overall biochemical processes and eventually to the growth of microorganisms. It is also suggested that, pH is more likely related to the transport of dye molecules across the cell membrane, which was considered as the rate limiting factor in decolorization performance (Lourenco et al., 2000[29]). To assess the maximum decolorization ability of consortium, it was tested against different concentrations of azo dyes (50 to 250 mg L^-1^). The complete decolorization of all the dyes was observed up to the concentration of 100 mg L^-1^ within 30 h at 30 ± 0.2 °C temperature and further increase in dye concentration resulted in reduction of decolorization efficiencies (Figure 2d(Fig. 2)). Lower decolorization rate by bacterial consortium at higher dyes concentration was reported (Phugare et al., 2011[41]). The decrease in decolorization efficiency at higher dye concentration may be due to the toxic effect of dye molecules on bacteria and/or less bacterial biomass for the uptake of higher dye concentration (Saratale et al., 2009[47]). Hence, 100 mg L^-1^ of all dyes concentrations were selected for further degradation and detoxification studies at microaerophilic, aerobic and sequential incubation processes. 

### Decolorization at various processes

The results of the decolorization study showed that, 98-99 % decolorization of all the four dyes was observed within 30 h of incubation under microaerophilic conditions (Table 2(Tab. 2)). However, variation in decolorization time was observed for all the dyes viz. RB 5 (30 h), RO 16 (12 h), DR 78 (18 h) and DR 81 (24 h) and that may be due to the structural differences of azo dyes used (Moosvi et al., 2005[35]). It has been reported that the azo dyes with mono azo bond were more likely to be decolorized faster than those with diazo or triazo groups (Franciscon et al., 2009[15]). For this reason, RO 16 and DR 78, which are both monoazo dyes showed a short decolorization time (12 and 18 h, respectively) and the highly substituted diazo RB 5 and DR 81 showed longer decolorization times (30 and 24 h, respectively). It is suggested that, the azo dyes with a hydroxyl or amino groups were methoxy, sulfo or nitro groups (Nigam et al., 1996[37]). Hence, in the present study variation in decolorization time was observed for all the dyes as these have difference in number of azo bonds and its position. The complete decolorization of all the dyes at microaerophilic conditions suggest that this process favors the decolorization and which may be due to the involvement of reductive enzymes in initial break-down of azo dyes. It was reported that, reductive cleavage of azo bonds (–N=N–) by azoreductase normally occurs in absence or reduced levels of oxygen as in microaerophilic conditions (Chang et al., 2001[5]; Sheth and Dave, 2009[51]). In contrast to the complete decolorization of all the four dyes under microaerophilic condition, the reduced performance only up to 12 % for RB 5, 20 % for RO 16, 22 % for DR 78 and 21 % for DR 81 was achieved under aerobic conditions within the same time and even no complete decolorization was observed in 48 h (Table 2(Tab. 2)). A previous study on aerobic decolorization of azo dyes has also shown that this process is insufficient to completely degrade most of azo dyes (Ogugbue et al., 2012[38]). 

Results of the sequential aerobic/micro-aerophilic and microaerophilic/aerobic process demonstrated that, bacterial consortium was able to completely decolorize all the four azo dyes within 30 h viz. RB 5 (98 % in 30 h), RO 16 (99 % in 12 h), DR 78 (98 % in 18h) and DR 81 (99 % in 24 h) (Table 2(Tab. 2)). These observations are in agreement with previous reports where potential of sequential aerobic/microaerophilic and microaerophilic/aerobic processes in the enhanced decolorization of azo dyes have been reported (Franciscon et al., 2009[15]; Waghmode et al., 2011[57], 2012[58]). It has been suggested that, sequential processes carried out the reductive and/or oxidative break-down of azo dyes which results in its complete decolorization (Waghmode et al., 2012[58]). The role of azoreductase enzymes in decolorization of azo dyes under microaerophilic conditions has been previously reported (Saratale et al., 2010[46]). Alternatively, aerobic degradation of textile azo dyes by bacterial consortium has been also reported (Senan and Abraham, 2004[48]; Tony et al., 2009[55]). However, the aerobic decolorization of azo dyes depends on the oxidation potential of microorganisms. Mixed bacterial culture SKB-II consisted of five bacterial strains as *Bacillus vallismortis*, *Bacillus pumilus*, *Bacillus cereus*, *Bacillus subtilis* and *Bacillus megaterium* exhibited good potential (43-71 %) in aerobic decolorization of azo dyes viz. Congo red, Bordeaux, Ranocid Fast and Blue BCC (Tony et al., 2009[55]). The involvement of H_2_O_2_ independent oxidase especially laccase in aerobic decolorization of azo dyes has been previously reported (Senan and Abraham, 2004[48]; Chivukala and Renganathan, 1995[10]). In context with these reports, the present bacterial consortium showed complete decolorization of all the azo dyes by both sequential processes as an initial reduction of azo bond in microaerophilic condition and/or oxidation under aerobic condition. 

### Formation of aromatic amines and TOC reduction

The general approach of biodegradation is to mineralize the textile dyes using natural capability of native microorganisms. But, most of the textile azo dyes are xenobiotics and its biodegradation results into formation of aromatic amines, which are more toxic than parent dyes and even carcinogenic and/ or mutagenic (Levine, 1991[28]). Formation of aromatic amines by bacterial degradation of azo dyes under microaerophilic conditions has been reported (Franciscon et al., 2009[15]). Aerobic treatments for biodegradation of azo dyes have also been reported; however, such oxidative break-down of azo bond is not sufficient to completely mineralize them into nontoxic form (Ogugbue et al., 2012[38]). Hence, in the present study the safe biode-gradation processes for complete mineralization of azo dyes without formation of aromatic amines have been evaluated. The broth obtained after decolorization under microaerophilic, aerobic and both sequential processes by using bacterial consortium was analyzed for presence of aromatic amines and TOC reduction. 

Spectrophotometric analysis data reveals the presence of aromatic amines in all the four azo dyes decolorized broths under microaerophilic conditions viz. RB 5 (0.26 mM), RO 16 (0.18 mM), DR 78 (0.20 mM) and DR 81 (0.23 mM) (Table 2(Tab. 2)). The formation of colorless aromatic amines in microaerophilic conditions can be a result of reductive cleavage of azo bond (–N=N–) (Chung et al., 1992[11]). This occurs due to the activity of azoreductase when carriers in the electron transport chain utilize azo dyes as terminal electron acceptors in absence of oxygen and results in the formation of aromatic amines (Singh et al., 2007[52]; Van der Zee and Villaverde, 2005[56]). In contrary, the aerobic process doesn’t show formation of aromatic amines, but very less (12 to 22 %) decolorization was observed within the same time. This suggests the inability of bacterial consortium for aerobic degradation of selected azo dyes. 

Results of the sequential aerobic/micro-aerophilic processes also showed formation of aromatic amines even after complete decolorization of all the azo dyes viz. RB 5 (0.08 mM), RO 16 (0.06 mM), DR 78 (0.07 mM) and DR 81 (0.09 mM). This is because of the initial oxidation of azo dyes which might be repressed by the absence of oxygen in further microaerophilic stage. Thus, aromatic amines resulting from the decolorization process under microaerophilic stage are not mineralized further by subsequent aerobic stage and thus tend to accumulate in the decolorized broth (Gottlieb et al., 2003[17]; Luangdilok and Panswad, 2000[32]). In contrast, sequential microaerophilic/aerobic processes completely decolorized all the dyes and did not show the presence of aromatic amines in dyes decolorized broth. These results indicate that the aromatic amines produced under microaerophilic conditions were ultimately removed in the aerobic stage suggesting its potential as safe process for biodegradation of azo dyes. It has been known that reductive enzymes such as azoreductase carried out the initial break-down of azo bonds in microaerophilic condition while oxidative enzymes such as laccase further performs the oxidative cleavage of formed amines in aerobic condition (Zimmerman et al., 1982[61]; Duran and Esposito, 2000[14]). These findings are in agreement with previous studies where sequential microaerophilic/aerobic processes capable of removing azo dyes via biotransformation and/ or biodegradation without forming aromatic amines have been reported (Franciscon et al., 2009[15]). In another report, a sequential anaerobic/aerobic system was used to treat a simulated textile wastewater containing azo dyes in which significant amount of aromatic amines was removed successfully in sequential aerobic stage (Isik and Sponza, 2008[20]).

The degradation of azo dyes by bacterial consortium was confirmed by TOC analysis. Result of the decolorization study under microaerophilic conditions showed 63, 68, 72, and 81 % reduction in TOC level for RB 5, RO 16, DR 78, and DR 81 respectively suggesting their mineralization (Table 2(Tab. 2)). Also, the bacterial consortium showed enhanced TOC reduction under sequential aerobic/microaerophilic processes viz. RB 5 (63 %), RO 16 (69 %), DR 78 (72 %) and DR 81 (65 %); and sequential microaerophilic/ aerobic process as well viz. RB 5 (62 %), RO 16 (68 %), DR 78 (72 %) and DR 81 (64 %). In contrast, under aerobic conditions the removal ratio for TOC was very less viz. RB 5 (10 %), RO 16 (20 %), DR 78 (20 %) and DR 81 (18 %). The significant reduction in TOC from dyes decolorized broth under microaerophilic as well as sequential processes supported the mineralization of azo dyes. Thus, the present bacterial consortium was found to be promising for biodegradation of azo dyes under microaerophilic and sequential processes. However, complete absence of aromatic amines with optimum reduction in TOC content was observed only under sequential microaerophilic/aerobic processes suggesting its potential in biodegradation and detoxification of textile azo dye contaminated sites.

### Enzyme analysis

Microbial consortium capable of decolorizing textile dyes via biotransformation and/ or biodegradation have been reported and the efficiency of degradation and detoxification are known to be dependent on the activities of enzymes produced by microorganisms in the consortiums (Pasti-Grigsby et al., 1992[40]; Senan and Abraham, 2004[48]). Thus, to understand the mechanism of azo dyes degradation by bacterial consortium which doesn’t produce aromatic amines under sequential microaerophilic/aerobic processes; the activities of oxidative (laccase and veratryl alcohol oxidase) and reductive (azoreductase and NADH-DCIP reductase) enzymes were monitored in dyes decolorized broth. 

Enzymatic activity analysis of bacterial consortium reveals the involvement of four studied enzymes viz. laccase, veratryl alcohol oxidase, azoreductase and NADH-DCIP reductase in biotransformation of selected azo dyes. Significant induction in the activities of reductive enzymes viz. azo reductase and NADH DCIP reductase by 97 and 229 % for RB 5, 55 and 160 % for RO 16, 63 and 196 % for DR 78, and 108 and 258 % for DR 81 respectively as compared to control (bacterial consortium without addition of dye) (Table 3(Tab. 3)). Higher induction in reductive enzymes after decolorization of all the four azo dyes supports the active role of bacterial consortium in the sequential biodegradation process. It is suggested that, the reductive cleavage of azo bond is the initial step in bacterial metabolism of azo dyes under microaerophilic conditions (Franciscon et al., 2009[15]). In agreement with these findings, our results also showed significant induction of azo reductase activities, suggesting the reduction of azo bonds. However, the single microaerophilic incubation condition reveals the presence of aromatic amines in all the dyes decolorized broth supporting that the reduction is the initial step in bacterial degradation of azo dyes (Table 2(Tab. 2)). The inductive pattern for NADH-DCIP reductase by a developed microbial consortium consisting of *Proteus vulgaris* NCIM-2027 and* Micrococcus glutamicus* NCIM-2168 during decolorization of textile azo dye Scarlet R was reported (Saratale et al., 2009[47]).

In contrary to induced reductases, the slight increase in oxidative enzyme activities of laccase and veratryl alcohol oxidase as 28 and 45 % for RB 5, 14 and 20 % for RO 16, 19 and 26 % for DR 78, and 36 and 51 % for DR 81 were respectively detected (Table 3(Tab. 3)). The less induction in oxidative enzyme activities might be due to the subsequent degradation of formed amines which were in fewer quantities than parent dyes. In the same contest, slight increase in activities of oxidative enzymes laccase and veratryl alcohol oxidase were previously reported in a fungal-bacterial consortium degrading textile azo dye Rubine GFL (Lade et al., 2012[27]). Therefore, in the present study, complete degradation of all azo dyes without forming aromatic amines was observed only under sequential microaerophilic/aerobic processes. The significant induction in reductase enzyme activities support the initial reduction of azo dyes under microaerophilic step and slight increase in oxidative enzyme activities suggest the further oxidation of formed amines under subsequent aerobic step. 

### Acute toxicity studies

It is of great concern to study the toxicity of azo dyes before and after degradation by microorganisms as such dyes as well as their degradation metabolites are known to be toxic for human health and aquatic biota (Chequer et al., 2009[8]; Lu et al., 2010[31]). The acute tests with *Daphnia magna* have been known as a first screening method for the evaluation of lethal toxicity of new chemicals to mammals and humans (Guilhermino et al., 2000[18]). Thus, the relative sensitivities of four azo dyes used and its degradation products after treatment with bacterial consortium were assessed by acute toxicity test with *Daphnia magna*. The test was carried out in 75 % of original azo dyes containing broth as 100 % mortality has been observed in the original as well as 25 and 50 % diluted broths. This is in agreement with a previous report, where complete mortality of *Daphnia magna* was found in original and 1:2 diluted azo dyes decolorized broths (Franciscon et al., 2009[15]).

The results of the toxicity assay are presented as percent death of *Daphnia magna* occurred in the bacterial consortium treated azo dyes broth under different incubation processes. The toxicity data showed that degradation under microaerophilic and sequential aerobic/microaerophilic conditions were not sufficient to remove the complete toxicity of dyes as little (1 to 4 %) mortality of *Daphnia magna* was observed in these treatment samples (Table 4(Tab. 4)). In addition, the partially decolorized azo dyes samples under aerobic conditions showed near about same mortality as like control dyes viz. 39 % RB5, 35 % RO16, 37 % DR78 and 41 % DR 81. In contrast, the sequential microaerophilic/ aerobic process treated dyes sample doesn’t show any mortality suggesting the complete removal of toxicity from decolorized broths. Therefore, a last sequential aerobic stage is necessary to diminish the toxicity from dyes decolorized broths. It can be concluded that, even bacterial consortium was able to completely decolorize all the dyes under microaerophilic, sequential aerobic/microaerophilic and sequential microaerophilic/aerobic processes, the final aerobic stage is necessary for aromatic amines removal (Sponza and Isik, 2005[53]). 

## Conclusions

All four tested textile azo dyes were completely decolorized by the bacterial consortium under microaerophilic, sequential aerobic/microaerophilic and microaerophilic/ aerobic processes with some differences in decolorization times depending on the dyes structures. The sequential microaerophilic/ aerobic process was found to be very effective in azo dyes decolorization as no aromatic amines detected in dye-decolorized broths. Additionally, significant reduction in the TOC content from all the dye-decolorized broths under this process suggested its efficacy for mineralization of dyes. The significant induction in the activities of azo reductase and NADH-DCIP reductase suggested the initial reduction of azo bonds under microaerophilic stage while slight increase in laccase and veratryl alcohol oxidase activities confirmed further oxidation of formed amines by subsequent aerobic stage. Acute toxicity test with *Daphnia magna* revealed complete detoxification of azo dye-degraded metabolites under sequential microaerophilic/aerobic processes. Thus, the sequential microaerophilic/aerobic process provides good evidence of the applicability of developed bacterial consortium as a promising and safe method in biodegradation and detoxification of azo dyes.

## Notes

Diby Paul (Department of Environmental Engineering, Konkuk University, Seoul-143-701, Korea, Phone: +82-2-450-3318; Fax: +82-2-450-3542) and Sanjay Govindwar (Department of Biochemistry, Shivaji University, Kolhapur-416004, India, Phone: +91-231-2609152; Fax: +91-231-2691533) contributed equally as corresponding authors.

## Conflict of interest

The authors declare no conflict of interest. 

## Figures and Tables

**Table 1:**
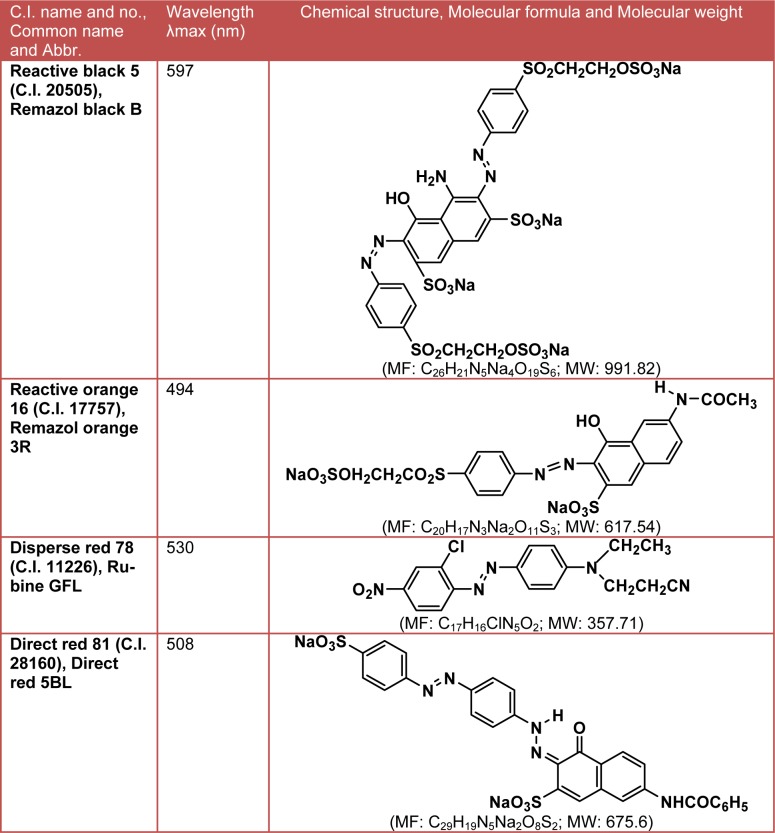
The structural information of textile azo dyes used in this study

**Table 2:**
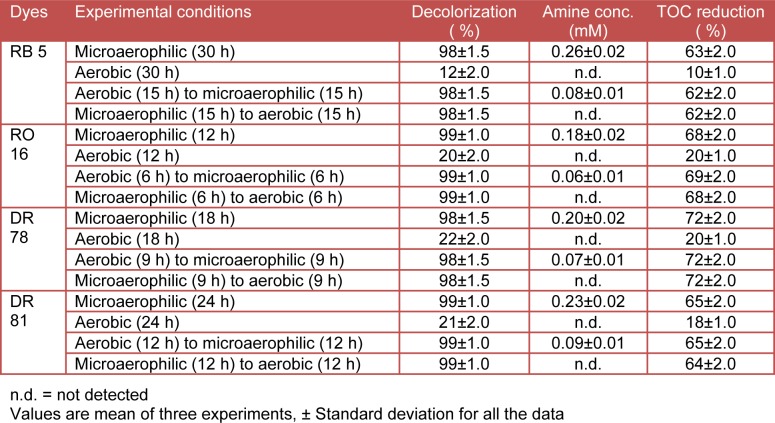
Decolorization of azo dyes by bacterial consortium and formation of aromatic amines and TOC reduction under various experimental conditions

**Table 3:**
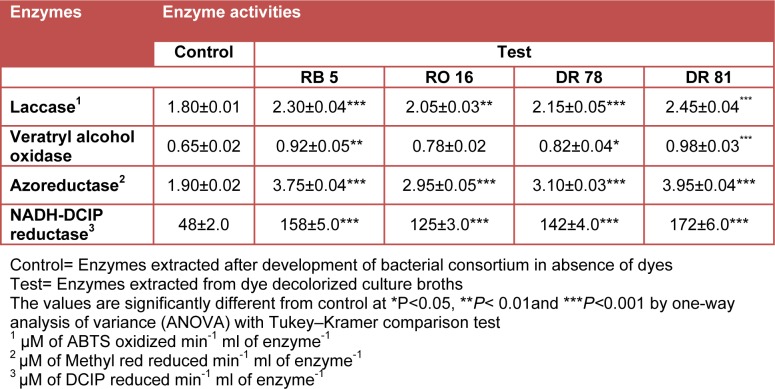
Enzyme activities after decolorization of azo dyes by bacterial consortium under sequential microaerophilic/aerobic processes

**Table 4:**
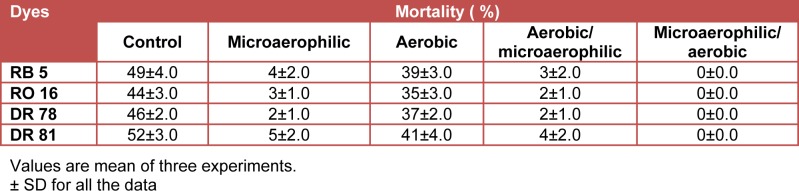
Mortality of *Daphnia magna* exposed to 75 % dilution of the culture supernatants containing azo dyes treated with bacteria consortium under different incubation processes

## References

[1] Ambrósio ST, Campos-Takaki GM (2004). Decolorization of reactive azo dyes by Cunninghamella elegans UCP 542 under co-metabolic conditions. Bioresour Technol.

[2] Asgher M, Bhatti HN, Shah SAH, Asad MJ, Legge RL (2007). Decolorization potential of mixed microbial consortia for reactive and disperse textile dyestuffs. Biodegradation.

[3] Banat IM, Nigam P, Singh D, Marchant R (1996). Microbial decolorization of textile-dye-containing effluents: a review. Bioresour Technol.

[4] Bumpus JA, Singh VP (1995). Microbial degradation of azo dyes. Biotransformations: Microbial degradation of health-risk compounds (pp 157-75)..

[5] Chang JS, Chou C, Lin YC, Lin PJ, Ho JY, Hu, TL (2001). Kinetic characteristics of bacterial azo-dye decolorization by Pseudomonas luteola. Water Res.

[6] Chen BY, Chang JS (2007). Assessment upon species evolution of mixed consortia for azo dye decolorization. J Chin Inst Chem Eng.

[7] Chen H, Hopper SL, Cerniglia CE (2005). Biochemical and molecular characterization of an azoreductase from Staphylococcus aereus, a tetrameric NADPH-dependent flavoprotein. Microbiology.

[8] Chequer FM, Angeli JP, Ferraz ER, Tsuboy MS, Marcarini JC, Mantovani MS (2009). The azo dyes Disperse Red 1 and Disperse Orange 1 increase the micronuclei frequencies in human lymphocytes and in HepG2 cells. Mutat Res.

[9] Cheunbarn T, Cheunbarn S, Khumjai T (2008). Prospects of bacterial granule for treatment of real textile industrial wastewater. Int J Agric Biol.

[10] Chivukula M, Renganathan V (1995). Phenolic azo dye oxidation by laccase from Pyricularia oryzae. Appl Environ Microbiol.

[11] Chung KT, Stevens SE, Cerniglia CE (1992). The reduction of azo dyes by the intestinal microflora. Crit Rev Microbiol.

[12] Correia VM, Stephenson T, Judd SJ (1994). Characterization of textile wastewaters - A review. Environ Technol.

[13] Dubey SK, Pandey A, Bajaj AK, Misra K (2007). Some commercial azo dyes as inhibitors of mushroom tyrosinase DOPA oxidase activity. J Pharmacol Toxicol.

[14] Duran N, Esposito E (2000). Potential applications of oxidative enzymes and phenoloxidase-like compounds in wastewater and soil treatment: a review. Appl Catal B Environ.

[15] Franciscon E, Zille A, Dias GF, Ragagnin MC, Durrant LR, Cavaco-Paulo A (2009). Biodegradation of textile azo dyes by a facultative Staphylococcus arlettae strain VN-11 using a sequential microaerophilic/aerobic process. Int Biodeterior Biodegr.

[16] Franciscon E, Zille A, Durrant LR, Fantinatti GF, Cavaco-Paulo A (2009). Microaerophilic-aerobic sequential decolourization/biodegradation of textile azo dyes by a facultative Klebsiella sp. Strain VN-31. Process Biochem.

[17] Gottlieb A, Shaw C, Smith A, Wheatley A, Forsythe S (2003). The toxicity of textile reactive azo dyes after hydrolysis and decolourisation. J Biotechnol.

[18] Guilhermino L, Diamantino T, Silva MC, Soares AM (2000). Acute toxicity test with Daphnia magna: an alternative to mammals in the prescreening of chemical toxicity. Ecotoxicol Environ Saf.

[19] Hu TL (2001). Kinetics of azoreductase and assessment of toxicity of metabolic products from azo dye by Psudomonas luteola. Water Sci Technol.

[20] Isik M, Sponza DT (2008). Anaerobic/aerobic treatment of a simulated textile wastewater. Sep Purif Technol.

[21] Jadhav JP, Parshetti GK, Kalme SD, Govindwar SP (2007). Decolourization of azo dye methyl red by Saccharomyces cerevisiae MTCC 463. Chemosphere.

[22] Jadhav UU, Dawkar VV, Tamboli DP, Govindwar SP (2009). Purification and characterization of veratryl alcohol oxidase from Comamonas sp. UVS and its role in decolorization of textile dyes. Biotechnol Bioprocess Eng.

[23] Kadam AA, Telke AA, Jagtap SS, Govindwar SP (2011). Decolorization of adsorbed textile dyes by developed consortium of Pseudomonas sp. SUK1 and Aspergillus ochraceus NCIM-1146 under solid state fermentation. J Hazard Mater.

[24] Kalyani DC, Patil PS, Jadhav JP, Govindwar SP (2008). Biodegradation of reactive textile dye red BLI by an isolated bacterium Pseudomonas sp. SUK1. Bioresour Technol.

[25] Kalyani DC, Telke AA, Dhanve RS, Jadhav JP (2009). Ecofriendly biodegradation and detoxification of Reactive Red 2 textile dye by newly isolated Pseudomonas sp. SUK1. J Hazard Mater.

[26] Khaled A, El Nemr A, El-Sikaily A, Abdelwahab O (2009). Removal of Direct N Blue-106 from artificial textile dye effluent using activated carbon from orange peel: Adsorption isotherm and kinetic studies. J Hazard Mater.

[27] Lade HS, Waghmode TR, Kadam AA, Govindwar SP (2012). Enhanced biodegradation and detoxification of disperse azo dye Rubine GFL and textile industry effluent by defined fungal-bacterial consortium. Int Biodeterior Biodegr.

[28] Levine WG (1991). Metabolism of azo dyes: implication for detoxification and activation. Drug Metab Rev.

[29] Lourenco ND, Novais JM, Pinheiro HM (2000). Reactive textile dye colour removal in a sequencing batch reactor. Water Sci Technol.

[30] Lowry OH, Rosebrough NJ, Farr AL, Randall RJ (1951). Protein measurement with the Folin phenol reagent. J Biol Chem.

[31] Lu K, Zhang XL, Zhao YL, Wu ZL (2010). Removal of color from textile dyeing wastewater by foam separation. J Hazard Mater.

[32] Luangdilok W, Panswad T (2000). Effect of chemical structures of reactive dyes on color removal by an anaerobic–aerobic process. Water Sci Technol.

[33] Marik J, Song A, Lam KS (2003). Detection of primary aromatic amines on solid phase. Tetrahedron Lett.

[34] Meyer U, Leisinger T, Cook AM, Hutter R, Nuesch J (1981). Biodegradation of synthetic organic colorants. Microbial degradation of xenobiotic and recalcitrant compounds..

[35] Moosvi S, Keharia H, Madamwar D (2005). Decolorization of textile dye ReactiveViolet 5 by a newly isolated bacterial consortium RVM 11.1. World J Microbiol Biotechnol.

[36] Moosvi S, Kher X, Madamwar D (2007). Isolation, characterization and decolorization of textile dyes by a mixed bacterial consortium JW-2. Dyes Pigm.

[37] Nigam P, Banat IM, Singh D, Marchant R (1996). Microbial process for the decolorization of textile effluent containing azo, diazo and reactive dyes. Process Biochem.

[38] Ogugbue CJ, Morad N, Sawidis T, Oranusi NA (2012). Decolorization and partial mineralization of a polyazo dye by Bacillus firmus immobilized within tubular polymeric gel. 3. Biotech.

[39] Pandey A, Singh P, Iyengar L (2007). Bacterial decolorization and degradation of azo dyes. Int Biodeterior Biodegr.

[40] Pasti-Grigsby MB, Paszczynski A, Goszczynski S, Crawford DL, Crawford RL (1992). Influence of aromatic substitution patterns on azo dye degradability by Streptomyces spp and Phanerochaete chrysosporium. Appl Environ Microbiol.

[41] Phugare SS, Kalyani DC, Patiol AV, Jadhav JP (2011). Textile dye degradation by bacterial consortium and subsequent toxicological analysis of dye and dye metabolites using cytotoxicity, genotoxicity and oxidative stress studies. J Hazard Mater.

[42] Phugare SS, Kalyani DC, Surwase SN, Jadhav JP (2011). Ecofriendly degradation, decolorization and detoxification of textile effluent by a developed bacterial consortium. Ecotoxicol Environ Saf.

[43] Pinheiro HM, Touraud E, Thomas O (2004). Aromatic amines from azo dye reduction: status review with emphasis on direct UV spectrophotometric detection in textile industry wastewaters. Dyes Pigm.

[44] Robinson T, McMullan G, Marchant R, Nigam P (2001). Remediation of dyes in textile effluent: a critical review on current treatment technologies with a proposed alternative. Bioresour Technol.

[45] Salokhe MD, Govindwar SP (1999). Effect of carbon source on the biotransformation enzyme in Serratia marcescens. World J Microbiol Biotechnol.

[46] Saratale RG, Saratale GD, Chang JS, Govindwar SP (2010). Decolorization and biodegradation of reactive dyes and dye wastewater by a developed bacterial consortium. Biodegradation.

[47] Saratale RG, Saratale GD, Kalyani DC, Chang JS, Govindwar SP (2009). Enhanced decolorization and biodegradation of textile azo dye Scarlet R by using developed microbial consortium-GR. Bioresour Technol.

[48] Senan RC, Abraham TE (2004). Bioremediation of textile azo dyes by aerobic bacterial consortium. Biodegradation.

[49] Sharma VK (2009). Aggregation and toxicity of titanium dioxide nanoparticles in aquatic environment - A Review. J Environ Sci Health A.

[50] Shaul GM, Holdsworth TJ, Demmpsey CR, Dostal KA (1991). Fate of water soluble azo dyes in the activated sludge process. Chemosphere.

[51] Sheth NT, Dave SR (2009). Optimisation for enhanced decolourization and degradation of Reactive Red BS C.I. 111 by Pseudomonas aeruginosa NGKCTS. Biodegradation.

[52] Singh P, Sanghi R, Pandey A, Iyengar L (2007). Decolorization and partial degradation of monoazo dyes in sequential fixed-filmed anaerobic batch reactor (SFABR). Bioresour Technol.

[53] Sponza DT, Isik M (2005). Toxicity and intermediates of CI Direct Red 28 dye through sequential anaerobic/aer-obic treatment. Process Biochem.

[54] Tan NCG, Lettinga GH, Field JA (1999). Reduction of the azo dye mordant orange 1 by methanogenic granular sludge exposed to oxygen. Bioresour Technol.

[55] Tony BD, Goyal D, Khanna S (2009). Decolorization of textile azo dyes by aerobic bacterial consortium. Int Biodeterior Biodegr.

[56] Van der Zee FP, Villaverde S (2005). Combined anaerobic–aerobic treatment of azo dyes—a short review of bioreactor studies. Water Res.

[57] Waghmode TR, Kurade MB, Khandare RV, Govindwar SP (2011). A sequential aerobic/microaerophilic decolorization of sulfonated mono azo dye Golden yellow HER by microbial consortium GG-BL. Int Biodeterior Biodegr.

[58] Waghmode TR, Kurade MB, Lade HS, Govindwar SP (2012). Decolorization and biodegradation of Rubine GFL by microbial consortium GG-BL in sequential aerobic/microaerophilic process. Appl Biochem Biotechnol.

[59] Wolfenden BS, Willson RL (1982). Radical-cations as reference chromogens in kinetic studies of ono-electron transfer reactions: pulse radiolysis studies of 2 2[prime or minute]-azinobis-(3-ethylbenzthiazoline-6-sulphonate). J Chem Soc Perkin Trans.

[60] Wong Y, Yu J (1999). Laccase-catalyzed decolorization of synthetic dyes. Water Res.

[61] Zimmerman T, Kulla HG, Leisinger T (1982). Properties of purified orange II azo reductase, the enzyme initiating azo dye degradation by Pseudomonas KF46. Eur J Biochem.

